# Selective Detection of Hydrogen Sulfide and Methane by a Single MOX-Sensor

**DOI:** 10.3390/s19051135

**Published:** 2019-03-06

**Authors:** Alexey Shaposhnik, Pavel Moskalev, Elena Sizask, Stanislav Ryabtsev, Alexey Vasiliev

**Affiliations:** 1 Department of Chemistry, Voronezh State Agrarian University, Voronezh 394087, Russia; a.v.shaposhnik@gmail.com (A.S.); hilda04091996@mail.ru (E.S.); 2 Department of Mathematics and Physics, Voronezh State Agrarian University, Voronezh 394087, Russia; 3 Research Institute of Physics, Voronezh State University, Voronezh 394006, Russia; ryabtsev@niif.vsu.ru; 4 NRC Kurchatov Institute, Moscow 123182, Russia; a-a-vasiliev@yandex.ru

**Keywords:** MOX sensor, temperature modulation, selectivity, sensitivity, qualitative analysis, multidimensional data

## Abstract

In this paper, we describe a technique for the qualitative and quantitative analysis of such gas mixtures as “hydrogen sulfide in air” and “methane in air” using temperature modulation of a single metal oxide sensor. Using regression analysis in the principal components plane (PC1, PC2), we performed a selective determination of analytes on the minimum set of their concentrations in the training set, which is essential for solving practical problems. An important feature of this work is the difference in test gas concentrations from their concentrations in the training set. For the qualitative analysis of gas mixtures in a wide range of concentrations, we have developed an improved method for processing arrays of multidimensional data. For this improvement, we form a Mahalanobis neighborhood for polynomial regression lines constructed from the projection of training samples for each analyte on the (PC1, PC2) plane. Using the temperature modulation mode for the metal oxide sensor allowed us to increase its response when determining hydrogen sulfide by two to four orders of magnitude compared with the constant temperature mode.

## 1. Introduction

Analysis of a gas medium can be performed with simple and compact devices based on metal oxide gas sensors (MOX sensors). The main disadvantage of MOX sensors is their low selectivity. All reducing gases lead to a decrease in the electrical resistance of *n*-type semiconductors; therefore, it is rather difficult to distinguish one analyzed reducing gas from another, for example, carbon monoxide from hydrogen. Meanwhile, the industry needs simple and compact instruments designed for the qualitative analysis of gas mediums, which can be considered as one-component. Such devices should, for example, distinguish between gas mixtures of “methane in the air” and “ethanol vapor in the air” with the minimum probability of false positives when determining methane. Another example is the need to distinguish between mixtures “methane in the air” and “hydrogen sulfide in the air”, which is important for some gas fields, where periodic emissions of hydrogen sulfide are observed.

To improve the selectivity of metal oxide semiconductor gas sensors, it is possible to use the following approaches:

- additives that increase the sensitivity to target gases [1];
- selective gas permeable membranes [2];
- systems that include many sensors that differ in cross-sensitivity to different gases (“electronic nose”) [3];
- temperature modulation of the MOX sensor.

Both special catalytic additives and selective gas-permeable membranes can slightly increase cross-sensitivity to the target gas, but this is not enough for proper qualitative analysis, so researchers have to use instruments known as “electronic noses”, which combine several low-selective sensors. Chemometric analysis of multidimensional data arrays obtained in such multisensor devices, in principle, can allow solving the problem of qualitative analysis, but devices of this type have significant limitations—as the number of sensors in the device increases, its instability grows exponentially. On the contrary, reducing the number of sensors allows not only increasing stability, but also reducing their power consumption, which is especially important for autonomous devices.

A more promising solution to the problem of qualitative analysis is pulsed temperature modulation of MOX sensors. In this mode, the amount of information about the gas medium increases because the processes of chemisorption of the reducing gas, its chemical reactions with chemisorbed oxygen and desorption of the reaction products can be separated in time, which allows us to identify the individual characteristics of analyte gases. However, the use of temperature modulation of metal oxide sensors still does not guarantee the correct solution to the problem of qualitative analysis. If the differences in the dependence of the sensor resistance on time in each measurement cycle are insignificant, then there is no possibility to distinguish one analyte gas from another. We need such compositions of gas sensitive layers and temperature modulation regimes, on which characteristic extremes would appear on the curves of resistance versus time in measuring cycles. The analysis of such extremes allows us to distinguish one gas analyte from another in a wide range of concentrations, which solves the problem of qualitative analysis.

Earlier, different regimes of temperature modulation were used by researchers. In most experiments, simple harmonic modulation of sensor temperature with a constant period [4,5,6,7,8,9,10,11,12,13,14,15] or modes close to them [16] was applied.

The application of sinusoidal temperature modulation presumes the pre-set of modulation parameters (period, temperature amplitude, and temperature base level). These parameters must be chosen from the set of virtual values, given their advantages and disadvantages. Because of this, the researchers used another approach, consisting of the application of the complex non-stationary rule, which was set by a pseudo-random oscillation generator [17,18,19]. The advantage of this approach is an increase in information about the gas medium. An important disadvantage is a very strong increase in time necessary for gas analysis.

In a number of works, the feedback regime was used for the measurements. In this case, for example, the temperature of the sensor was controlled in a way to maintain the constant value of the sensing layer of the sensor [20,21,22].

Instead of the smooth sinusoidal regime of sensor temperature modulation, it is possible to use fast impulse heating and relatively fast cooling down [23,24,25,26,27]. The application of an impulse heating regime enables the saving of heating power and, respectively use an autonomous power source. In addition, this approach permits an increase in the efficiency of qualitative gas analysis.

An optimal combination of the composition of the gas sensing layer and a regime of temperature modulation is a necessary but not sufficient condition for the solution of the problem of qualitative gas analysis. It is necessary also to carry out a correct chemometric analysis of the arrays of multidimensional data. In many types of research, the methods enabling the reduction of the number of dimensions of initial multidimensional data, that is compression of information were used. For this purpose, researchers used the Fourier transform [4,8,9,10,11,12,13,14,15], discrete wavelet transform [5,6,7,8], principal component analysis [25], artificial neural networks [16], and combinations of these methods. These transforms permit the principle demonstration of the possibility of qualitative analysis, but the procedure of this analysis have not been formalized in a correct way according to the requirements of analytical chemistry. The experimental values in two or three-dimensional space corresponding to different concentrations were not consolidated into a single area related to a target gas or, in other cases, this area was formed arbitrarily, without taking into account dispersion of data entering the learning data set, or the area corresponding to the target gas did not have a common border with the area corresponding to clean air.

In many studies on the selective determination of gases using low-selective chemical sensors, the gas concentrations in the tests coincided with the gas concentrations in the training sample. In these studies, the researchers demonstrated success in the qualitative determination of both individual gases and their mixtures, but in reality they identified the composition of predefined gas mixtures for a predetermined discrete set of analyte gas concentrations. The qualitative analysis of gas mixtures at concentrations different from the values in the training sample was not considered at all in these papers. This approach played an important role at the initial stage of development of sensory methods in analytical chemistry, but it is not suitable for solving practical problems, because it requires calibration for all gas mixtures at all possible concentrations of analyte gases. In our opinion, it is precisely the complexity of the calibration that makes it impossible to create reliable and compact devices for the selective analysis of gases. The approach proposed in this paper allows us to create a selective gas analyzer suitable for qualitative and quantitative analysis, but not requiring very complex and lengthy calibration. We used this approach for gas analysis using a single-sensor system, but a similar approach can be used to process data from multi-sensor systems (“electronic noses”).

## 2. Experimental Methods

In our earlier work [25], we tested the impulse temperature regimes for gas sensing layers of different composition. However, results applicable to the qualitative analysis of such gas as hydrogen were obtained only using “classical” sensing material—tin dioxide decorated with palladium or decorated with palladium and platinum. Thus, the choice of the composition of the gas sensing layer was dictated by the concrete analytical task.

For the fabrication of the gas sensing layer of the sensors, we used tin dioxide nanopowder obtained from stannic acid. For this, we added ammonia solution to the solution of tin acetate (+4) in glacial acetic acid:

(1) $$\mathrm{Sn}{\left({\mathrm{CH}}_{3}\mathrm{COO}\right)}_{4}+4{\mathrm{NH}}_{3}+3H_{2}O⟶H_{2}{\mathrm{SnO}}_{3}↓+4{\mathrm{CH}}_{3}{\mathrm{COONH}}_{4}.$$

Stannic acid was separated from the solution in the centrifuge, washed by deionized water, dried and calcinated. As a result we obtained nano-powder of tin dioxide [25]:

(2) $$H_{2}{\mathrm{SnO}}_{3}\overset{t}{⟶}{\mathrm{SnO}}_{2}+H_{2}O.$$

The picture of the resulting tin dioxide powder was obtained using the Jeol JEM 1011 transmission electron microscope (TEM) operating at 100 kV (tungsten cathode). X-ray powder diffraction (XRD) study was performed using the BRUKER D8 ADVANCE diffractometer.

As a dopant, we used a solution of the tetraamminopalladium nitrate (+2). To get a paste, the powder consisting of a mixture of tin dioxide powder with dopant was mixed in a mortar with the viscous vehicle, a solution of ethylcellulose in terpineol. This ink was deposited as a thin layer onto a substrate containing platinum electrodes and platinum microheater. After sensing layer deposition, the substrate was dried and annealed at 1023 K. At this temperature, fragile gel with a high specific area was formed. Palladium from the complex compound was reduced at this temperature, final palladium content in the sensing material was of 3 wt.%.

The structure of the surface of the sensing layer was studied using the Jeol 6610 scanning electron microscope (SEM). Phase composition was investigated using XRD analyzer PANalytical (${\mathrm{CuK}}_{\alpha 1}$-radiation).

The study of the gas response of the sensor was carried out at a constant gas flow equal to $200\;{\mathrm{cm}}^{3}\;\min^{-1}$. We used mixtures of gas-analytes with synthetic air, certified according to specification No. 6-16-2956 [28], developed by D. I. Mendeleyev Institute for Metrology (VNIIM) for calibration of gas analyzers. Constant relative gas humidity equal to 33% was reached by bubbling of a part of gas flow through distilled water; this part of gas flow was saturated with water to the humidity of close to 100%. The flow rate were controlled using Bronkhorst F-111BI-1k0 mass flow controllers.

The microhotplate with a sensing layer was fabricated using thick film technology. The size of the sensor was of 2.8mm×0.5mm×0.1 mm, it is suspended in a TO-8 package with four Pt wires having 20 µm in diameter and about 3 mm long. The area of the sensing layer is of about 0.3mm×0.3 mm. According to our previous results, the temperature is uniform within ±10 K over the surface of the sensing layer. Thermal response time of the sensor that is characteristic time necessary to heat the sensing layer to desired temperature was of about 1 s. The details of thick film sensor design were given in [29].

The sensor was placed into the gas chamber made of stainless steel. Sensor temperature was measured using platinum microheater as a resistive temperature sensor. The resistance of this microheater was of about 10 Ohm at room temperature with temperature coefficient of resistance being of 0.28% per Kelvin. A specially developed electronic controller was able to measure the resistance of the sensing layer with frequency up to 40 Hz, and to control, as well, the temperature regime of the sensor.

If the sensor operated in constant temperature regime, it was kept at 573 K. If the sensor operated in a temperature modulation regime, the heating cycle duration was of 15 s, first 2 s of this period corresponded to the heating up to 723 K, whereas final 13 s corresponded to the cooling and stabilization to 373 K. In the latter case, the power consumed by a single sensor, averaged over the measuring cycle, was about 150 mW.

At the fabrication of microhotplates used in this work, special care was taken to avoid any thermal stresses in the sensor chip and to obtain sufficient temperature uniformity over the chip. The tests of long-term stability of the chip operating in constant temperature and in pulsing heating mode showed that the drift of the heater resistance at constant temperature did not exceed 3% per year at working temperature of 723 K, and the sensor withstood at least 5 million switch on-off cycles [24,29]. The sensor response was calculated as a ratio of the sensor resistance in gas medium under investigation *R* to the resistance in pure air $R_{0}$:

(3) $$S\;=\;R/R_{0}.$$

## 3. Results and Discussion

### 3.1. Sensing Material Characterization

**Figure 1:**
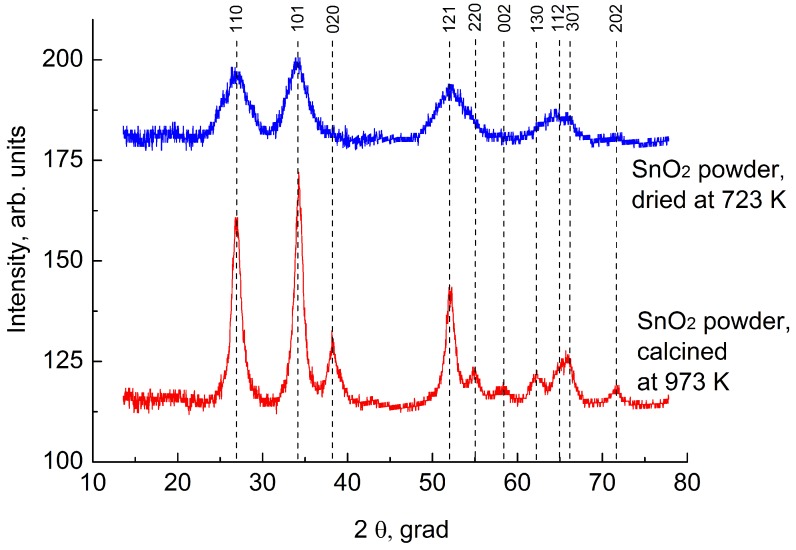
X-ray diffraction picture of the tin dioxide powder after dried at 723 K and calcined at 973 K.

**Figure 2:**
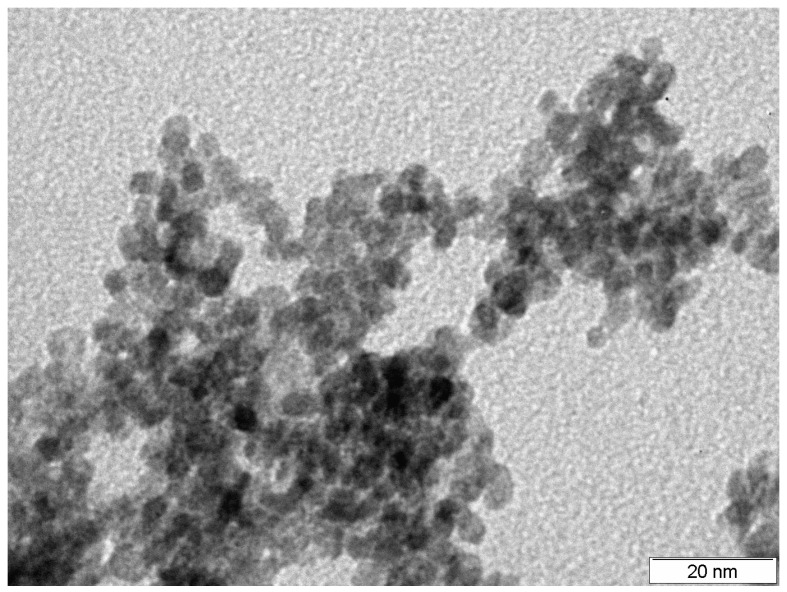
Tin dioxide powder image obtained on a transmission electron microscope.

Using the XRD analysis in Figure 1 we have shown that tin dioxide powder has a rutile structure. The red line corresponds to the tin dioxide powder dried at 723 K, and blue line to the powder calcined at 973 K. The width of the lines becomes smaller after annealing; this corresponds to the crystallite growth at the powder annealing. The powder after annealing was studied using TEM. Figure 2 shows that the average particle size is 4–5 nm.

**Figure 3:**
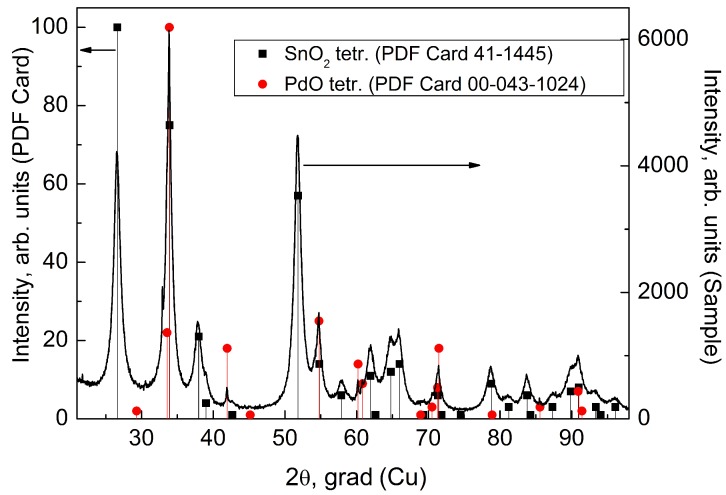
X-ray diffraction picture of the gas-sensitive layer.

The XRD method was used for the study of the phase composition of the final gas sensing layer. Figure 3 presents the XRD picture with superposed powder diffraction files (PDF) cards of ${\mathrm{SnO}}_{2}$-tetr. [30] and PdO-tetr. [31]. Normalized intensities of reflexes for PDF cards are marked on the left ordinate axis, whereas the intensity of the sample reflexes are marked on the right axis.

**Figure 4:**
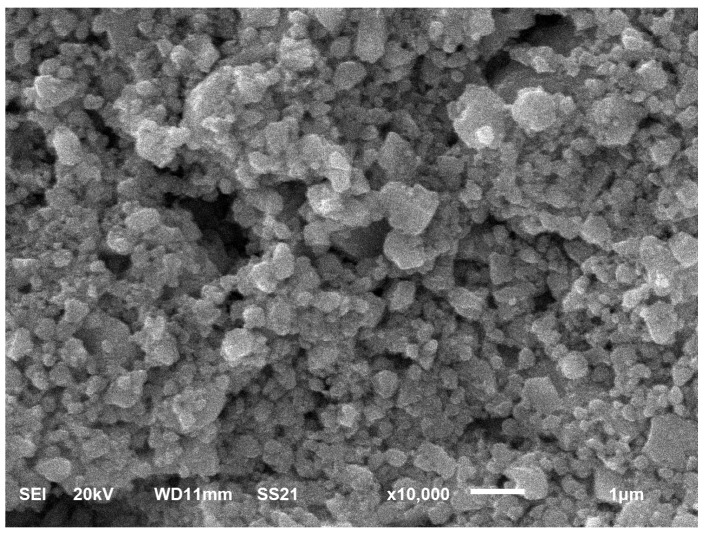
Gas-sensitive layer image obtained with a scanning electron microscope.

All X-ray reflexes were identified. In agreement with our assumption, the most intensive lines correspond to the tetragonal ${\mathrm{SnO}}_{2}$ phase. The intensity of reflexes of the PdO phase is much lower, because PdO content in the sample is of ∼3 wt.%. This is close to the detection limit of the XRD method. In addition, the most intensive reflexes of tetragonale PdO are superposed to the analogous reflexes of ${\mathrm{SnO}}_{2}$, for example at $33.8^{{}^{\circ}}$, $41.9^{{}^{\circ}}$, $54.8^{{}^{\circ}}$ angles. It is possible to identify the PdO phase only using reflexes at $42^{{}^{\circ}}$, $60.2^{{}^{\circ}}$, $60.8^{{}^{\circ}}$ angles, where they don’t overlape with the lines of the ${\mathrm{SnO}}_{2}$ phase. The microstructure of the gas sensing layer studied using SEM Jeol 6610 is presented in Figure 4.

### 3.2. Study of Gas Responses

The experiment at constant temperature of 573 K was carried out in the usual way. We determined the resistance of the sensing layer in synthetic air $\left(R_{0}\right)$ and in the gas mixture under investigation (R), and calculated the sensor response *S* using Formula (3). The experiment in a pulsing heating regime consisted in the measurement of sensing layer resistance during periodic heating cycles; the duration of each cycle was of 15 s, as it was described above.

**Figure 5:**
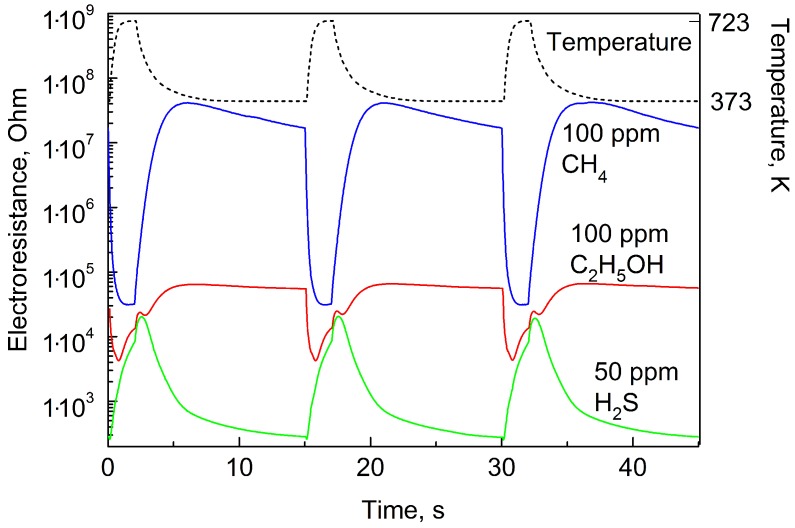
Temperature and electrical resistance of the sensor as a function of time during three measurement cycles at the determination of ${\mathrm{CH}}_{4}$, $C_{2}H_{5}\mathrm{OH}$ and $H_{2}S$ concentrations.

The dashed line in Figure 5 demonstrates the character of temperature change during three measurement cycles; blue line—change of sensing layer resistance at the concentration of ${\mathrm{CH}}_{4}$ (methane) equals to 100 ppm; red line—$C_{2}H_{5}\mathrm{OH}$ (ethanol vapor) concentration equals to 100 ppm; green line—$H_{2}S$ (hydrogen sulfide) concentration equals to 50 ppm.

Due to the correct choice of temperature cycling regime in combination with a proper choice of the sensing material, the character of curves corresponding to different gases is considerably different. This difference makes possible the selective determination of the concentration of hydrogen and other gases in a wide range of concentrations.

**Figure 6:**
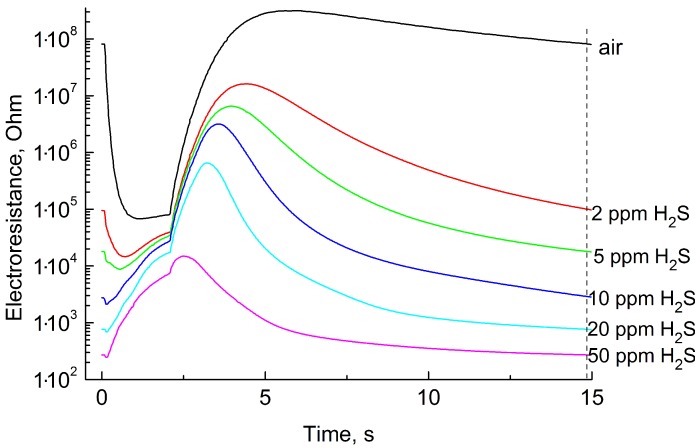
Electrical resistance of the sensor as a function of time during one measurement cycle at determining different $H_{2}S$ concentrations.

Figure 6 shows the sensing layer resistance as a function of time during one measurement cycle in clean air, 2, 5, 10, 20, and 50 ppm $H_{2}S$ (hydrogen sulfide).

All curves are non-monotonic and differ significantly from the black curve, which corresponds to clean air.

**Figure 7:**
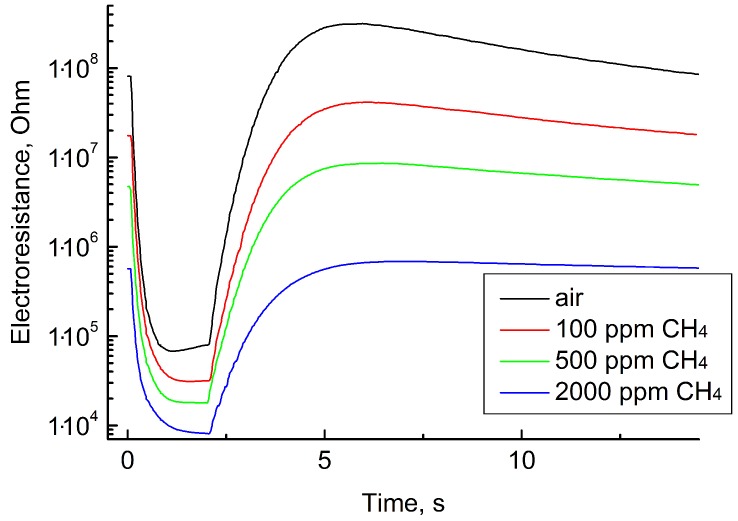
Electrical resistance of the sensor as a function of time during one cycle of measurements at determining different ${\mathrm{CH}}_{4}$ concentrations.

Figure 7 shows the sensing layer resistance as a function of time during one measurement cycle in clean air, 100, 500, and 2000 ppm ${\mathrm{CH}}_{4}$ (methane).

**Figure 8:**
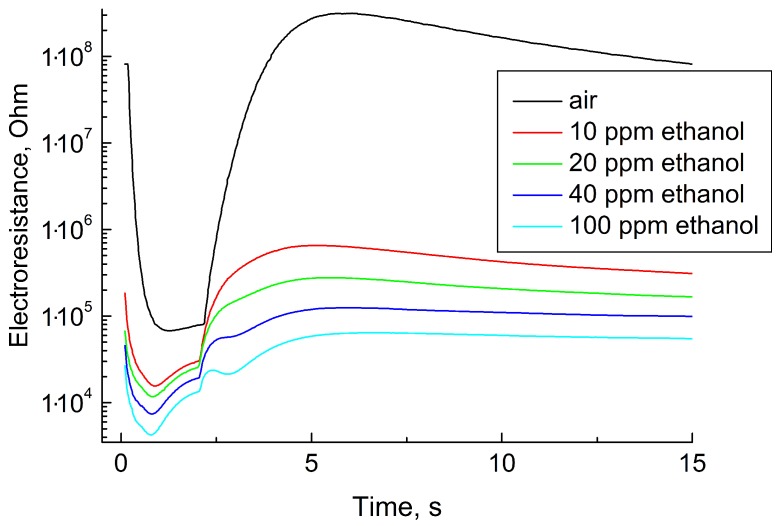
Electrical resistance of the sensor as a function of time during one cycle of measurements at determining different $C_{2}H_{5}\mathrm{OH}$ concentrations.

Figure 8 shows the sensing layer resistance as a function of time during one measurement cycle in clean air, 10, 20, 40, and 100 ppm $C_{2}H_{5}\mathrm{OH}$ (ethanol vapor). All curves are non-monotonic and differ significantly from the black curve, which corresponds to clean air. The shape of these curves is also significantly different from the curves obtained in the air mixed with hydrogen sulfide and methane.

### 3.3. Qualitative Analysis

We investigated the dependence of sensor resistance on time for at least 27 repeated measurement cycles for each concentration of hydrogen and other gases. Each cycle with a duration of 15 s and a sampling frequency of about 38 Hz generates 575 resistance values characterizing this gas system. Thus, each point of gas concentration corresponded to a point of 575-dimensional sample space. The results we obtained were a multidimensional data array, by which we first determined the minimum number of latent factors sufficient for a qualitative analysis of the gas mixture. Using the principal component analysis, we showed that the effective dimensionality of the data array can be reduced to two by the loss of less than 1% of the sample variance. Such a procedure is often used for multisensory systems (“electronic noses”), but in itself, it is not sufficient for correct qualitative analysis.

Formally, this procedure is realized by transforming the space of observable values $X\;=\;(x_{1},x_{2},\ldots ,x_{n})$ to the space of their linear orthonormal combinations $Z\;=\;(z_{1},z_{2},\ldots ,z_{n})$ that satisfy the dispersion monotonicity condition $D\left(z_{1}\right)\geq D\left(z_{2}\right)\geq \ldots \geq D\left(z_{n}\right)$. The linear combinations $z_{i}$ of the observed values $x_{i}$, called principal components (PC), will be sorted according to the information significance [32]. Since among all linear orthonormal combinations, the first principal component $z_{1}$ has the largest variance, it will contain the most significant features of the sample. Then the second principal component of $z_{2}$ will contain the following information-relevant features of the sample.

Then, for the data included in the training sample and projected onto the plane of the first two principal components, we constructed polynomial regression models that approximated by continuous lines the points corresponding to different concentrations of the same analyte. In this paper, to describe each analyte, we used third-order polynomial models linear with respect to their coefficients:

(4) $$z_{2i}\;=\;b_{0}+b_{1}z_{1i}+b_{2}z_{1i}^{2}+b_{3}z_{1i}^{3}+e_{i},$$

where $i\;=\;1,2,\ldots ,n_{k}$—indices and the number of training sample points for one analyte; $\left(z_{1i},z_{2i}\right)$—point coordinates for the training sample on the plane of the first pair of principal components; $b_{1}$, $b_{2}$, $b_{3}$—coefficients of the polynomial regression equation; $e_{i}$—deviations for the training sample points from the regression curve minimized by the least squares method $\sum_{i=1}^{n}e_{i}^{2}\to \;\min$.

**Figure 9:**
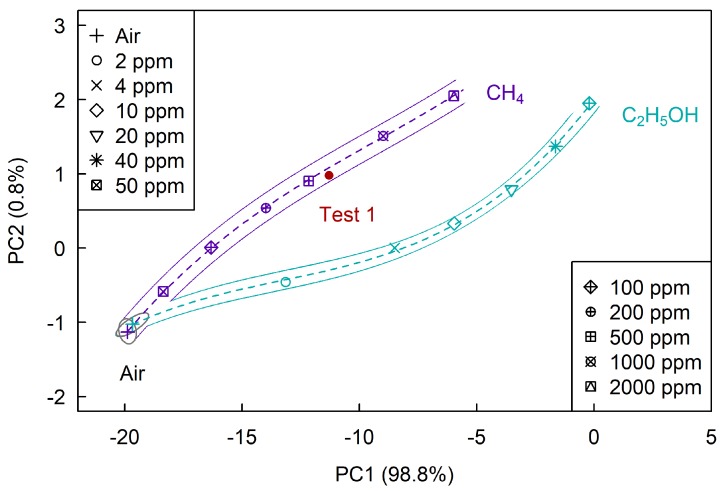
The (PC1, PC2) plane at the determination of ${\mathrm{CH}}_{4}$ and $C_{2}H_{5}\mathrm{OH}$.

**Figure 10:**
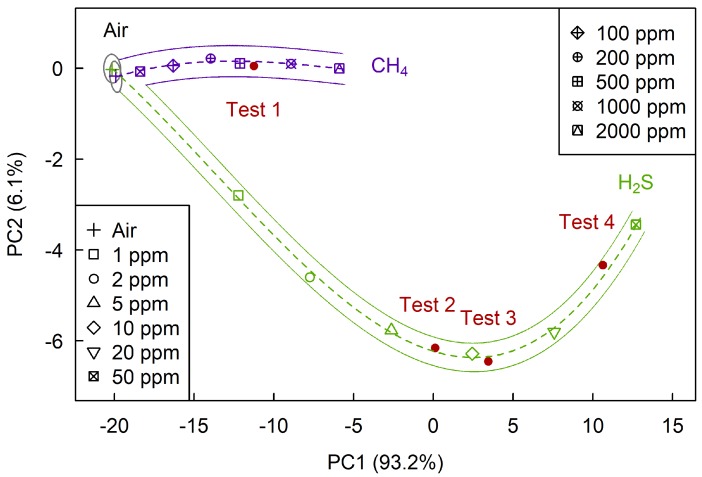
The (PC1, PC2) plane at the determination of $H_{2}S$ and ${\mathrm{CH}}_{4}$.

**Figure 11:**
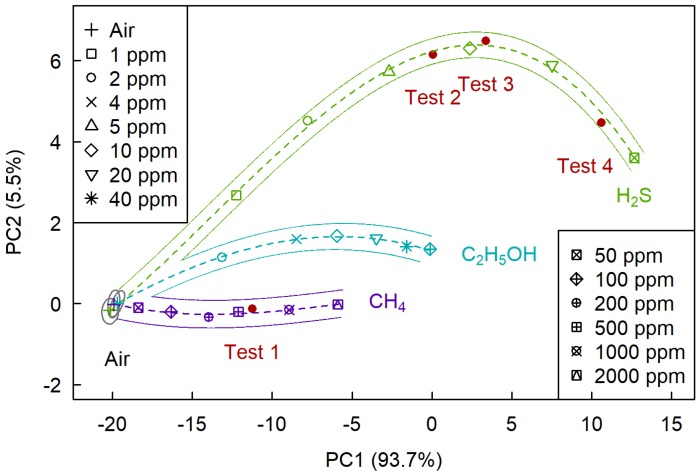
The (PC1, PC2) plane at the determination of $H_{2}$, ${\mathrm{CH}}_{4}$, and $C_{2}H_{5}\mathrm{OH}$.

Based on the regression curve and sample variances, we calculated the largest Malalanobis neighborhoods corresponding to each of the analytes. We determined these neighborhoods using the scale-invariant Mahalanobis distance, which, in addition to the deviations, takes into account the correlation between random vectors [33,34]:

(5) $$d\left(z_{a},z_{b}\right)\;=\;{\left({\left(z_{a}-z_{b}\right)}^{T}S^{-1}\left(z_{a}-z_{b}\right)\right)}^{-1/2}\leq \rho ,$$

where $z_{a}$, $z_{b}$ is an arbitrary point and a point belonging to the regression curve on the plane of the first two principal components; $S^{-1}$—inverse covariance matrix of the training sample subset corresponding to the analyte and approximated by the regression curve (4); $\rho \;=\;6\sigma_{\max}$—radius of the largest Mahalanobis neighborhood, determined by the largest standard deviation $\sigma_{\max}$ of the training sample subset in the direction from $z_{a}$ to $z_{b}$. In Figure 9, Figure 10 and Figure 11 these neighborhoods have the form of ellipses, whose translation along the regression lines forms sections for each of the analytes. The doubled radius of the confidence interval (compared to a typical radius of $\rho \;=\;3\sigma_{\max}$) allows us to lower the a priori estimate for the probability of a normal sampling point beyond the interval by six orders of magnitude (from $2.7\times 10^{-3}$ to $2.0\times 10^{-9}$), which is important for training samples larger than thousands of measuring cycles.

Figure 9 shows the plane of the first two principal components, on which we determined sections corresponding to air, methane and ethanol. The projection of sample data on the (PC1, PC2) plane contains about 99.6% of the sample variance, which is a very satisfactory result. As follows from the figure, selective determination of methane is possible at concentrations above 100 ppm and ethanol vapor—at concentrations above 2 ppm.

Figure 10 shows the plane of the first two principal components, on which we determined sections corresponding to air, hydrogen sulfide and methane. The projection of sample data on the (PC1, PC2) plane contains about 99.3% of the sample variance, which is also a very satisfactory result. As follows from the figure, selective determination of hydrogen sulfide, in this case, is possible at concentrations above 1 ppm and methane—at concentrations above 100 ppm.

Figure 11 shows the plane of the first two principal components, on which we determined sections corresponding to air, hydrogen sulfide, methane, and ethanol. As follows from the figure, selective determination of hydrogen sulfide, in this case, is possible at concentrations above 1 ppm, ethanol—at concentration above 2 ppm, and methane—at concentrations above 100 ppm.

During the experiment, we also obtained test data corresponding to three different concentrations of hydrogen sulfide and one concentration of methane that were not included in the training sample. Test data we transformed into the space of the principal components, determined by training samples. Formally, this corresponds to the matrix multiplication of the test point vectors by the eigenvectors of the principal components [35]. After that, the problem of qualitative analysis is reduced to the question of how far are the projections of the test points from the Mahalanobis neighborhoods near the regression lines corresponding to each of the analytes.

As shown in Figure 9, Figure 10 and Figure 11 all four test points belong to the section corresponding to their analytes, which tells us that the problem of selective determination of hydrogen sulfide and methane was correctly solved.

### 3.4. Quantitative Analysis

To determine the response of sensor operating in the regime of temperature modulation, it is possible to use, in principle, any value of resistance during the measurement cycle. However, it is reasonable to use a value corresponding to the moment, where the response is highest. For the measurement of hydrogen sulfide concentration, we used the values of resistance obtained 14.5 s after the beginning of each cycle (this moment is marked by a dashed line in Figure 6). At the quantitative analysis of other gases, it is reasonable to use the values of resistance obtained at other moments, where the response to the appropriate gas is highest. The choice of the optimal value of resistance is possible due to the preliminary procedure of qualitative analysis.

**Figure 12:**
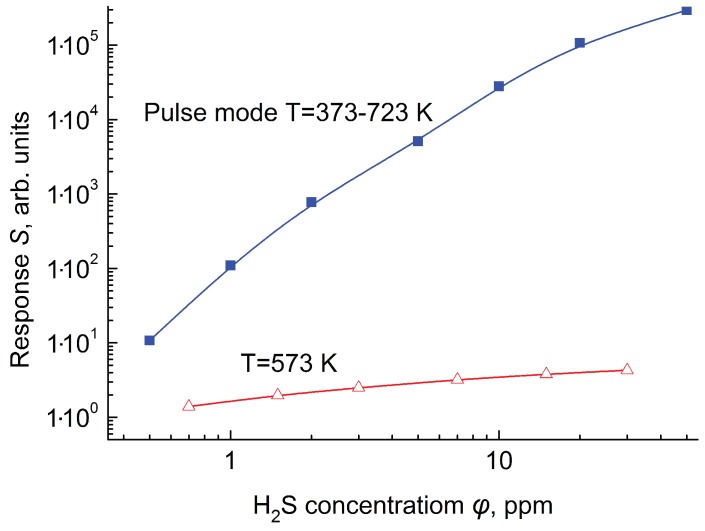
Sensor response as a function of $H_{2}S$ concentration for constant and modulation temperature mode.

The red line on Figure 12 shows the values of the response of the sensor operating at the constant temperature equal to 573 K, and the blue line the values of the response of the sensor operating in modulation mode with temperatures from 373 to 723 K. This plots demonstrate that the substitution of constant temperature operation regime with temperature modulation regime leads to an increase in sensor response by two–four orders of magnitude and to respective increase in sensor sensitivity in a range from 5 to 50 ppm (this range is of particular interest for the instruments used for early discovery of fire and for medical application of sensor).

When choosing a constant sensor temperature T=573 K for hydrogen sulfide in the air, we took into account that the maximum sensor response can be obtained at lower temperatures (about 450 K), but at such temperatures the relaxation time of the gas sensitive layer will increase significantly, which will increase the required duration measuring cycle.

What is the reason of the increase in gas sensitivity of the sensors operating in temperature modulation mode? From our point of view, a decrease in temperature of the gas sensitive layer leads to an increase in sorption of the gas analyte. However, at low temperature, these molecules are not activated and the chemical reaction with these molecules can not occur. At high temperature, hydrogen sulfide molecules are activated and become to be able to participate in chemical reactions involving oxygen anions $O_{2}^{-}$, $O^{-}$ or $O^{2-}$ adsorbed on the surface of the semiconductor sensing layer. On the other hand, an increase in temperature leads to a decrease in sorption of a gas analyte. Therefore, to measure gas response at the constant temperature, it is necessary to find a compromise: an increase in temperature reduces sorption of gas, but a decrease in temperature decreases catalytic activity of the sensing layer and decreases the number of activated molecules. Also, as the temperature decreases, a significant increase in the relaxation time will be observed. Note that after the hydrogen sulfide concentration in the air drops to zero (to pure air), the resistance of the sensor will very slowly return to its original values, because the desorption of hydrogen sulfide and the products of its chemical interaction with oxygen is difficult.

Pulsed heating of semiconductor sensors allows us to combine the advantages of two temperature ranges of the sensor. At the beginning of the measurement cycle, the temperature of the sensor is low and gas sorption reaches maximum. Sensor heating up to high temperature (723 K) activates the catalyst and enables the hydrogen sulfide molecule activation. These activated molecules can interact with an excess of oxygen anions. Fast heating of the sensing layer means that hydrogen sulfide molecules can be activated and can interact with oxygen anions on the surface before their desorption would take place. This mechanism explains a strong increase in sensor response operating in a pulsing heating mode with fast heating/cooling. Such an increase in sensor response has not been observed at the application of sinusoidal temperature modulation.

## 4. Conclusions

The combination of a specially adjusted temperature regime of sensor operation with an optimized composition of the sensing layer enabled the solution of several important problems. First, metal oxide semiconductor gas sensor, which is characterized initially by low selectivity, permitted us to perform a correctly qualitative analysis of hydrogen sulfide and methane in the air. Second, the sensitivity of the gas sensor at the measurement of hydrogen sulfide concentrations increase up to very high values. Third, the application of the non-stationary temperature regime enables a decrease in power consumption of the gas sensor; this makes possible the application of such sensors in miniaturized gadget able to work using small batteries.

In this paper, we show the solution of an important chemometric problem—the qualitative and quantitative analysis of conventionally one-component systems “hydrogen sulfide in the air” and “methane in the air” using the minimum amount of training data for test concentrations of analytes that are not included in the training set. This approach can be used for processing data of multi-sensor systems and can be extended to solve more complex problems, such as determining the composition of multi-component gas mixtures.
